# Self‐sustained actuation from heat dissipation in liquid crystal polymer networks

**DOI:** 10.1002/pola.29032

**Published:** 2018-04-27

**Authors:** Ghislaine Vantomme, Anne Helene Gelebart, Dirk Jan Broer, E. W. Meijer

**Affiliations:** ^1^ Institute for Complex Molecular Systems (ICMS), Technical University of Eindhoven, 5600 MB Eindhoven The Netherlands; ^2^ Department of Chemical Engineering and Chemistry Laboratory of Macromolecular and Organic Chemistry, Technical University of Eindhoven, 5600 MB Eindhoven The Netherlands; ^3^ Department of Chemical Engineering and Chemistry, Laboratory for Functional Organic Materials and Devices (SFD) Technical University of Eindhoven, 5600 MB Eindhoven The Netherlands

**Keywords:** adaptive materials, liquid crystal polymer networks, liquid‐crystalline polymers, networks and stimuli‐sensitive polymers, oscillatory motion, photo‐thermal effect, soft robotics

## Abstract

Liquid crystal polymer networks (LCNs) lead the research geared toward macroscopic motion of materials. These actuators are molecularly programed to adapt their shape in response to external stimuli. Non‐photo‐responsive thin films of LCNs covered with heat absorbers (e.g., graphene or ink) are shown to continuously oscillate when exposed to light. The motion is governed by the heat dissipated at the film surface and the anisotropic thermal deformation of the network. The influence of the LC molecular alignment, the film thickness, and the LC matrix on the macroscopic motion is analyzed to probe the limits of the system. The insights gained from these experiments provide not only guidelines to create actuators by photo‐thermal or pure photo‐effects but also a simple method to generate mechanical oscillators for soft robotics and automated systems. © 2018 The Authors. Journal of Polymer Science Part A: Polymer Chemistry Published by Wiley Periodicals, Inc. J. Polym. Sci., Part A: Polym. Chem. **2018**, *56*, 1331–1336

## INTRODUCTION

Smart materials able to macroscopically move in response to environmental fluctuations and perform mechanical work is a topic of growing interest in polymer science. A multitude of examples of polymers performing a movement between two thermodynamically stable states upon an external stimulus have been disclosed in the last decades.^1^, ^2^, ^3^, ^4^ However, in the ongoing search for more complex functions inspired by nature, the challenge is to develop materials working out‐of‐equilibrium.^5^, ^6^, ^7^ So far most of the successes in autonomous movement of materials are based on the Belousov–Zhabotinsky (BZ) reaction as the chemical oscillating event used in gels.^8^, ^9^ Recently, Aida and coworkers published an intriguing system based on layered Π‐stacked carbon nitride polymer replying to small variations of humidity showing another unique way to create continuous motion. In that example, constant light irradiation induces desorption of water leading to fast bending, remarkable jump and directional “walking” of the polymeric film.^10^

Liquid crystal networks are identified as a pervasive class of polymeric materials to create photo‐actuators and self‐sustained oscillators.^11^ The macroscopic deformation obtained (curling, bending, swelling, etc.) is programed in the organization of the mesogens at the molecular level.^3^, ^12^, ^13^, ^14^, ^15^, ^16^ By playing with factors such as the liquid crystalline phase, the molecular alignment and the incorporation of photo‐responsive dyes (typically azobenzenes), a large variety of photo‐actuators have been described, working in equilibrium and out‐of‐equilibrium conditions.^17^, ^18^, ^19^, ^20^, ^21^ It is now well accepted that in glassy LCN, the trans‐to‐cis photo‐isomerization of the azobenzenes under UV irradiation leads to collective molecular geometrical changes translated to the macroscopic scale. Recently, the use of azobenzenes with fast thermal cis‐to‐trans back‐relaxation in combination with the creation of a feedback loop based on self‐shadowing has created fast and self‐sustained motion of polymeric film^22^, ^23^, ^24^ and shows promising applications for transport and self‐cleaning surfaces. The motion is governed by a thermal effect and the photo‐responsive azo‐derivatives dyes can be replaced by any component able to quickly dissipate light into heat, for example, dyes or photo‐stabilizers,^22^ or inorganic particles.^25^ The heat dissipated from the photo‐excited dopants throughout the LCN induces anisotropic changes in the dimensions of the network by thermal deformation and a large bending of the film. When the sweet spot between large bending, self‐shadowing and thermal oscillation of the film is obtained, self‐sustained oscillatory motion can be achieved.

Herein, we extend our previous work on continuous motion of LCNs^22^ to show that the simple deposition of a heat absorber (e.g., charcoal or ink) at the surface of an inactive LCN leads to self‐sustained oscillations. First, we explore the limits of the system to obtain continuous motion with a photo‐thermal effect by using a light absorbing photo‐stabilizer embedded in the LCN. The influence of the molecular alignment, the film thickness, and the storage modulus of the LC matrix is analyzed. From the knowledge gained in this study on the heat conduction in the LCN, the limit of the technical usability of the system is pushed further and we demonstrate that the surface deposition of heat absorber at the localized zone on the inactive LCN is sufficient to observe out‐of‐equilibrium motion. These results provide not only guidelines to create actuators by photo‐thermal or pure photo‐effects but also a simple method to generate mechanical oscillators.

## EXPERIMENTAL

### Materials and Mixture Preparation

The mesogens **D3**, **D6**, and **M6** were purchased at Merck. The mesogens **D11** was custom synthesized by Synthon and the mesogen **M11** and **M3** were provided by Philips research. The photo‐stabilizer Tinuvin 328 **1** and the photo‐initiator, Irgacure 819, were purchased at Ciba Specialty Chemicals. All materials were used without further purification. The mixtures were prepared by solubilizing the mesogens, photo‐stabilizer and photo‐initiator in about 1 mL of dichloromethane. The solvent was subsequently evaporated under a flow of argon.

### Cell Preparation and LCN Film Preparation

The cell of 20 µm‐thick gap was prepared by gluing together two glass plates coated with either a planar alignment layer (Polyimide Optimer Al 1501, JSR corporation, Japan), or a homeotropic alignment layer (Polyimide Sunever grade 5300). The planar plates are rubbed to direct the molecules into a single direction. According the alignment needed, the cells are either made of two planar glass plates placed at 180° with respect to the rubbing direction (uniaxial planar alignment), two planar glass plates placed at 90° with respect to the rubbing direction (twist alignment), two homeotropic glass plates (uniaxial homeotropic alignment) or one planar and one homeotropic glass plates (splay alignment). The cells are filled at the isotropic temperature of the mixture and polymerized in their nematic phase under UV light source (EXFO Omincure‐ S2000) for 10 min followed by a post curing at 130 °C. A full and detailed description of the procedure is reported.^26^

### LCN Characterization

The absorbance and transmittance of the LCN films were analyzed by UV–vis–NIR spectrophotometry (Shimadzu UV‐3102). The phase transition of each mixture was characterized by differential scanning calorimetry (DSC) on a TA instrument Q1000 equipment and by polarized optical microscopy (POM) performed on a Leica CTR600. The DSC measurements were performed at a rate of 5 °C/min, and the transitions are reported form the second cooling run. The dynamic mechanical thermal analysis (DMTA) was performed on a TA instrument‐Q800 equipment in a multi‐frequency‐strain mode with a frequency of 1 Hz.

### LCN Actuation

The LCNs were actuated with light‐emitting diode (LED) purchased at ThorLabs. The LEDs were mounted with a collimator to focus the light at a specific spot on the sample. The motion was recorded with a high speed camera (PCO 5.5 sCMOS) and the temperature was followed with a thermal camera (Xenics, Gobi‐640‐GigE).

## RESULTS AND DISCUSSION

### Influence of the Molecular Alignment on the Film Bending: Preamble to Oscillation

**Figure 1:**
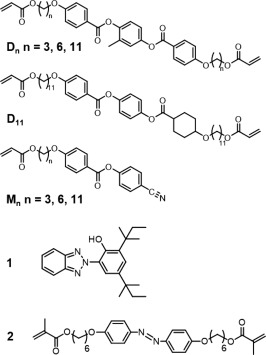
Molecular structures of the liquid crystal hosts (**Dn**, **D11**, and **Mn**), the photo‐stabilizer **1** and the azobenzene **2**.

**Figure 2:**
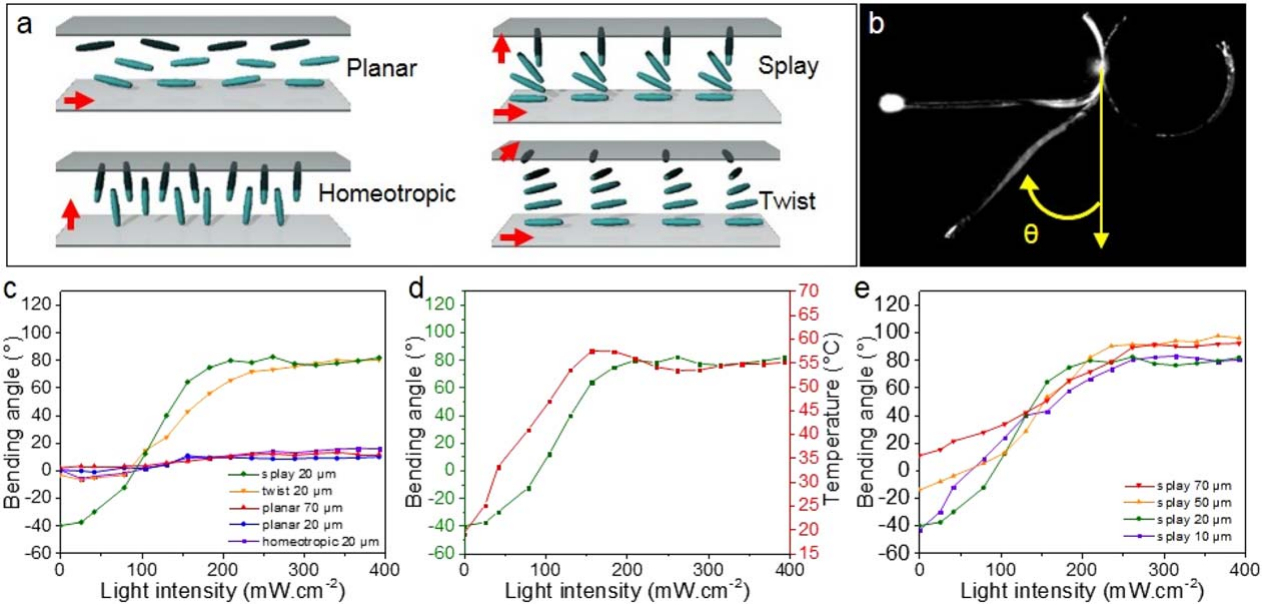
(a) Schematic representation of the LC alignments. The red arrows represent the molecular director. (b) Overlay of three pictures showing different bending angles and representation of the coordinate system used to measure the angle θ (in yellow). (c) Bending angles over the light intensity for different film alignments. (d) Temperature increase and bending angles upon incremental light intensity of a 20 µm splay aligned sample. (e) Bending angles over the light intensity for different film thicknesses of splay aligned films. The error on the angle measures was about ±5°. [Color figure can be viewed at http://wileyonlinelibrary.com]

We prepared LCNs by the photo‐polymerization of a mixture of commercially available mesogens RM82 **D6** (60 wt %) and RM23 **M6** (37.5 wt %), photo‐stabilizer Tinuvin **1** (2.5 wt %) and photo‐initiator (<1 wt %) as previously reported (Fig. 1).^22^, ^26^ Prior to polymerization, the mesogens were aligned in planar, homeotropic, splay, and twist fashion by the use of polyimide alignment layers, over the cross section of 20 µm [Fig. 2(a)]. The films obtained were cut in strips of about 2.5 cm long and 0.4 cm wide. Upon irradiation of the film with a UV source (365 nm, 0.5 W cm^−2^), the uniaxial aligned samples (planar and homeotropic) gave rise to small bending (less than 20°) while films with a gradual rotation of the molecular director throughout the film cross section (splay and twist) exhibited large and fast deformation (∼90°) [Fig. 2(b,c)].^27^ The motion originates from the photo‐excitation of the photo‐stabilizer **1**, which dissipates heat and subsequently induces thermal deformation of the LCN. The deformations of the twist and splay aligned samples were progressive over incremental light intensity until a plateau was reached at about 90°, that is, where the film is oriented with his long axis parallel to the light beam. The temperature at the position of the light spot, further denoted as the hinge, was monitored and exhibited a similar trend with a plateau at about 55 °C in the rubber state [Fig. 2(d)]. It is assumed that this plateau comes from a widening of the irradiated surface upon bending, which induces a local decrease of the light intensity. For comparison, a linear variation between temperature and light intensity was obtained when a constrained film was irradiated with increasing light intensity (see Supporting Information Fig. S1).

The UV–vis transmission spectra of the films were measured (see Supporting Information Fig. S2) and showed that light is transmitted through the 20 µm thin samples for the four alignments tested. In the case of the splay and twist alignments, the photo‐excitation of **1** over the whole thickness induced opposite thermal deformation at each side of the film, which resulted in a contraction at the planar side and an expansion at the homeotropic side, responsible for the large bending. However, for the uniaxial aligned sample (planar and homeotropic), complete excitation through the sample thickness led to the uniform contraction on both sides and consequently to a restricted bending. Interestingly, when two 20 µm films aligned planar and homeotropic were glued together to form a bilayer sample and irradiated, they displayed a bending of about 90° with a light intensity of 0.5 W cm^−2^. This large deformation is similar to the one observed for splay aligned sample. This result confirms that the opposite thermal deformation of the two layers: contraction at the planar layer and expansion at the homeotropic layer led to a complementary strain deformation and large bending.

As expected and described earlier,^16^ when the dopant Tinuvin **1** was replaced by the photo‐switch azobenzene **2** (Fig. 1) in a 20 µm planar film, large bending (∼90°) was observed and temperatures up to 55 °C were measured upon irradiation with UV light. Here, the trans‐to‐cis isomerization of the dye at the film surface induces the actuation and dominates the uniform thermal deformation by the heat transfer. To suppress the thermal effect, the film was submerged in water and irradiated. A large bending was still observed for the planar sample doped with the azobenzene **2**, while the splay samples containing the stabilizer **1** did not move. These results show that actuation of thin planar samples requires the use of photo‐switches, while for LCNs with a gradual transition of the molecular director throughout the film cross section, the photo‐thermal effect is sufficient.

### Influence of the Molecular Alignment on the Film Oscillation

**Figure 3:**
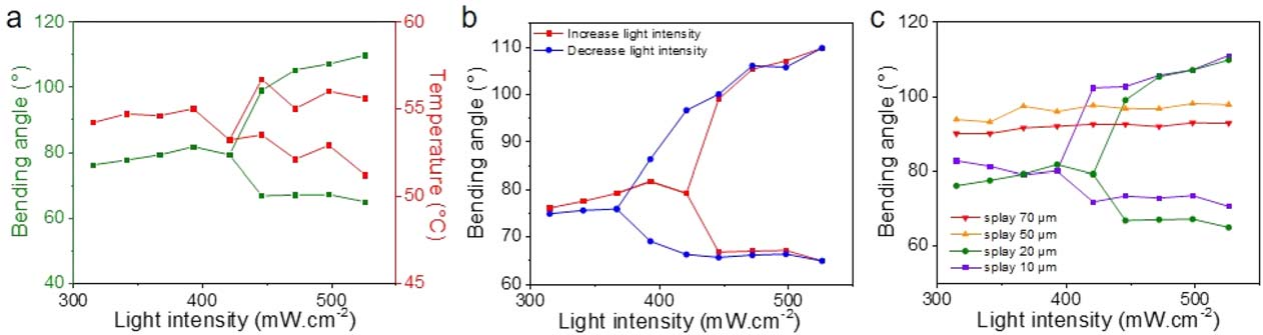
Bending angles and oscillations over the light intensity above 0.4 W cm^−2^ for splay aligned sample. (a) Temperature increase and bending angles upon incremental light intensity (the branched lines represent the amplitude of the mechanical and thermal oscillations). (b) Bending angles over the increase and decrease of the light intensity showing hysteresis. (c) Bending angles over the light intensity for different film thicknesses. The error on the amplitude of the mechanical oscillations is about ±5°. The measures close to the bifurcation points had a larger error of about ±10°. [Color figure can be viewed at http://wileyonlinelibrary.com]

Above 0.4 W cm^−2^, these samples showed mechanical and thermal oscillations between two maxima represented by the branched lines in Figure 3, with a frequency explained by the mechanics of a cantilever subjected to free vibration.^22^ In bifurcation theory, the branched point is seen as a local pitchfork bifurcation and indicates a change of stability of the system.^28^

Upon incremental light intensity, the amplitude of the oscillations increased while the frequency did not vary. At a light intensity of 0.5 W cm^−2^, the amplitudes of the mechanical and thermal oscillations reached about 40° and 3–4 °C, respectively [Fig. 3(a)]. Interestingly, the on/offset of oscillations was observed at different light intensities when the power of the lamp was ramping up or down. The pitchfork bifurcation point shifted to lower intensities when the lamp power was turned down presumably due to the film inertia [Fig. 3(b)]. However, the position of these pitchfork bifurcation points was highly dependent on the starting conditions of the motion, the amplitude of the oscillations, the position of the light and the shape of the film. When the light was switched off, oscillations stopped immediately which indicates a very high damping and a large energy dissipation. Moreover, at intensities between 0.3 and 0.4 W cm^−2^, damped oscillations were sometimes observed.

### Influence of the Film Thickness on the Film Motion

A thicker planar film of 70 µm was prepared to increase the light gradient through the film cross section, which could result in the preferential contraction of the exposed side and large deformation. However, although the light was totally absorbed by **1** through the film thickness (see Supporting Information Fig. S2), no bending was observed. Based on the heat diffusion equation, the estimated heat transfers across the thickness of the thin film is a few milliseconds (see SI). It means that the light gradient obtained cannot be associated with a similar heat gradient because of the quick heat conduction over the thin film, addressing both sides of the sample.

The influence of the film thickness was also evaluated on the deformation of the splay aligned samples [see Figs. 2(e) and 3(c)]. We reported earlier that the 20 µm splay aligned sample gave large deformations and steady oscillations under light irradiation. A systematic study on the effect of the length on the oscillatory motion has been performed earlier and was not duplicated here.^20^

The focus was rather placed on the thickness of the sample to understand how it affects the oscillation. To perform this study, films with thicknesses of 10, 20, 50, and 70 µm were prepared. Each film was irradiated with UV light (365 nm) at various intensities and exhibited large bending [Fig. 2(e)]. This observation supports our earlier comment on the fast heat transfer through the film thickness despite a partial light excitation. However, only the thinner samples oscillate [10 and 20 µm, Fig. 3(c)].

A decrease in film thickness reduces the light intensity threshold to obtain self‐sustained motion.^20^ This result suggests that the stiffness of the beam is a critical parameter to control the oscillations. The thicker samples are more resistant to deformation and they might require more light energy to reach the oscillatory regime.

This set of experiments proved that the geometrical dimensions as well as the choice of alignments are crucial parameters in order to achieve oscillations with this system. As a general rule to obtain oscillation by photo‐thermal effect, a gradient in the molecular director of the LC and a thin film geometry seem to be required.

### Influence of the LC Matrix on the Film Motion

We were curious to understand why the oscillation start at a threshold of temperature of 55 °C. To gain insights into this observation, we studied the influence of the LC matrix on the deformation. It was formerly reported that LCNs containing longer flexible aliphatic spacer have a lower glass transition temperature (*T_g_*) and a lower modulus due to an increase of the network mobility.^29^ Here, we prepared 20 µm thin splay aligned films with Tinuvin **1** (2.5 wt %) and 37.5 wt % of mono‐acrylate (**Mn**) and 60 wt % of diacrylate (**Dn**) mesogens, where n corresponds to the number of carbons (3, 6 and 11) in the aliphatic spacer (Fig. 1).

**Figure 4:**
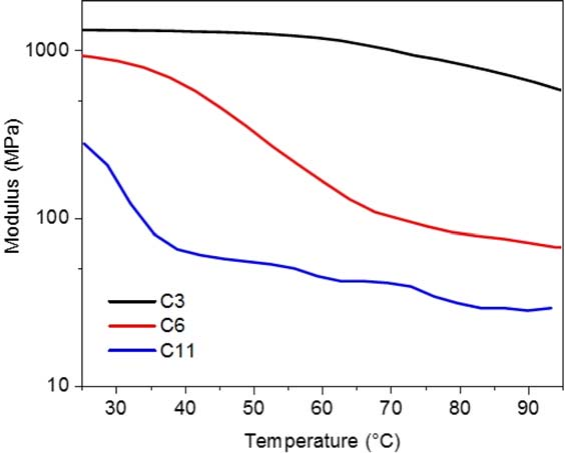
Storage modulus of the 20 µm splay aligned samples C3, C6, and C11 containing 2.5 wt % of Tinuvin **1** as a function of temperature during 365 nm LED irradiation. [Color figure can be viewed at http://wileyonlinelibrary.com]

The films were cut in thin strips (0.4 cm) and exposed to 365 nm LED light. They exhibited large bending but only the samples with the longer spacers (6 and 11 carbons) gave rise to oscillations in the range of light intensities investigated (<0.5 W cm^−2^). To understand the influence of the mechanical properties on the motion obtained, the samples were clamped in a DMTA and illuminated with increasing light power. The storage moduli of the material are reported as a function of the temperature reached during irradiation (Fig. 4). At room temperature, with no illumination, the storage moduli of the samples are about 1.3, 0.9, and 0.3 GPa for the matrices with the spacers C3, C6, and C11, respectively. Interestingly, upon irradiation (0.5 W cm^−2^), the films C6 and C11 attained the rubbery phase (*E* < 100 MPa) while the film C3 remained glassy (*E* > 600 MPa). Coupled to the fact that only the films C6 and C11 display self‐sustained motion, this result indicates that (at least) the hinge of the film should be rubbery to obtain oscillation, but not necessary to obtain simple bending.

Noteworthy, sample C6 (red trace in Fig. 4) started to oscillate when reaching the rubbery state but the C11 film (blue trace in Fig. 4) entered only the oscillatory regime at 55 °C, that is, well above the *T_g_* (40 °C). Based on the bending angle measured and correlated with the temperature in Figure 2(d), the bending angle of the film at 40 °C is −20°, which is not sufficient to obtain the self‐shadowing effect and the mechanism of oscillation. These results show that the combination of low storage modulus and large bending has to be obtained to reach self‐sustained motion.

### Oscillation of LCN by Surface Deposition of Heat Absorber

The knowledge gathered during this systematic study on the photo‐thermal effects and the heat conduction in the LCN prompted us to investigate the actuation of films by photo‐excitation of active compounds present only at the surface of a nondoped LCN. A splay aligned film (20 µm) was prepared as described above except that no dopant was added in the LC mixture prior to polymerization. The films obtained do not absorb light above 300 nm and upon UV light illumination no significant bending was observed (<10°). Charcoal was deposited on the surface to cover the entire film and remained stuck to the film by electrostatic interactions. The film was subsequently irradiated with LED 365 nm and displayed oscillatory motion with a frequency of 6.4 ± 0.3 Hz and amplitude of 27° ± 5°, which is in line with the previous results obtained with LCN film containing a dissolved heat absorber (Supporting Information Movie S1). Moreover, temperatures up to 64 °C were registered during the oscillation. Heat generated at the film surface by the photo‐excitation of the active compound is transported by conduction through the film thickness and actuates both sides of the film. This result confirms that the generation of the photo‐thermal effect at the surface is sufficient to actuate the film by heat conduction. This process is easily reversible by cleaning the film with a tissue to remove the charcoal and the film could be reuse numerous times.

**Figure 5:**
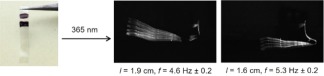
Pictures of the film covered with two lines of black marker (left). Overlay of pictures (right) from Supporting Information Movies S2 and S3 taken during oscillation. [Color figure can be viewed at http://wileyonlinelibrary.com]

Then, as previously applied to shape memory polymers,^30^ two lines were drawn with a black marker on the inactive film at 1.6 and 1.9 cm (Fig. 5). The film was placed into the beam of the LED 365 nm. When the light was scanned on the film, it bent precisely and solely at the position of the line and started to continuously oscillate with a frequency of 4.6 ± 0.2 Hz (with a film length 1.9 cm, Supporting Information Movie S2) and of 5.3 ± 0.2 Hz (with a film length 1.6 cm, Supporting Information Movie S3). This result confirms both our previous statement that surface activation and a localized active zone^22^ is sufficient to bring the system in an oscillatory regime.

## CONCLUSIONS

In conclusion, we report the bending and oscillatory motion of thin LCN actuators under light irradiation by simple deposition of heat absorbers on the film surface. In particular, these experiments show the prerequisites to obtain oscillatory motion by a pure thermal effect: a gradual rotation of the molecular director of the mesogens in the network throughout the film cross section, a thin thickness and a low modulus when the sample is heated by light irradiation. The results described show the ease to transform an inactive thin LCN film into an oscillator, with a precise control of the oscillations frequency depending on the location of the heat absorbers on the surface.

This research will impact grandly the community of photo‐induced actuation in LCN since pure heat effects at the surface appear to be sufficient to obtain deformations such as bending and oscillations. This general postactuation strategy is a versatile method to easily access out‐of‐equilibrium motion of LCN and to pattern the surface in multitude fashion to obtain complex motion.^31^

## Supporting information

Supporting Information Movie S1

Supporting Information Movie S2

Supporting Information Movie S3

Supporting Information Figures
