# Supplementary material for: Self‐sustained actuation from heat dissipation in liquid crystal polymer networks
Source: J Polym Sci A Polym Chem. 2018 Apr 27;56(13):1331–6. doi: 10.1002/pola.29032 (PMC6001434; doi:10.1002/pola.29032)
Supplement: Supplementary file 4 — Supporting Information Figures [file POLA-56-1331-s004.pdf]

Supporting Information

Self-sustained actuation from heat dissipation in liquid crystal polymer networks

Ghislaine Vantomme,[1,2*] Anne Helene Gelebart,[1,3*] Dirk J. Broer,[1,3] E.W. Meijer[1,2]

1 Institute for Complex Molecular Systems (ICMS) - Technical University of Eindhoven, 5600 MB Eindhoven, The Netherlands

2 Department of Chemical Engineering and Chemistry- Laboratory of Macromolecular and Organic Chemistry-Technical University of Eindhoven, 5600 MB Eindhoven, The Netherlands

3 Department of Chemical Engineering and Chemistry- Laboratory for Functional Organic Materials and Devices (SFD)-Technical University of Eindhoven, 5600 MB Eindhoven, The Netherlands

Correspondence to: Ghislaine Vantomme, Dirk J. Broer and E. W. Meijer (E-mail: g.vantomme@tue.nl, d.broer@tue.nl, e.w.meijer@tue.nl)

[*] These authors contributed equally to the work.

Movie S1. Photo-induced self-oscillating motion of a splay aligned film (20 µm) covered with charcoal, clamped on one side and irradiated with a LED (365 nm, 510 mW.cm^{-2}) from the left side.

Movie S2. Photo-induced self-oscillating motion of a splay aligned film (20 µm) clamped on one side and irradiated with a LED (365 nm, 510 mW.cm^{-2}) from the left side. The light beam reaches the film at a length of 1.9 cm where a black line is drawn on the film.

Movie S3. Photo-induced self-oscillating motion of a splay aligned film (20 µm) clamped on one side and irradiated with a LED (365 nm, 510 mW.cm^{-2}) from the left side. The light beam reaches the film at a length of 1.6 cm where a black line is drawn on the film.

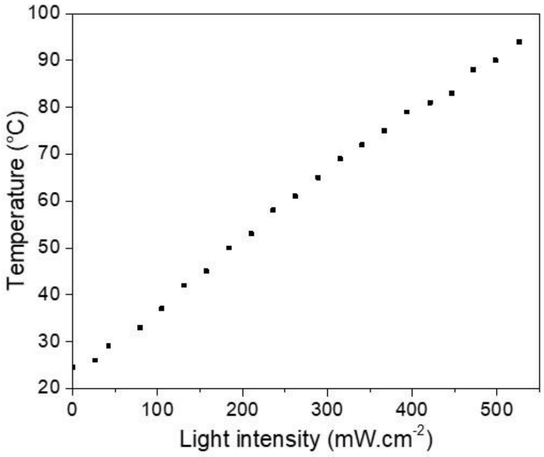

Figure S1. Temperature increase upon incremental light intensity of a constrained 20 µm splay aligned sample (the sample cannot move during the experiment).

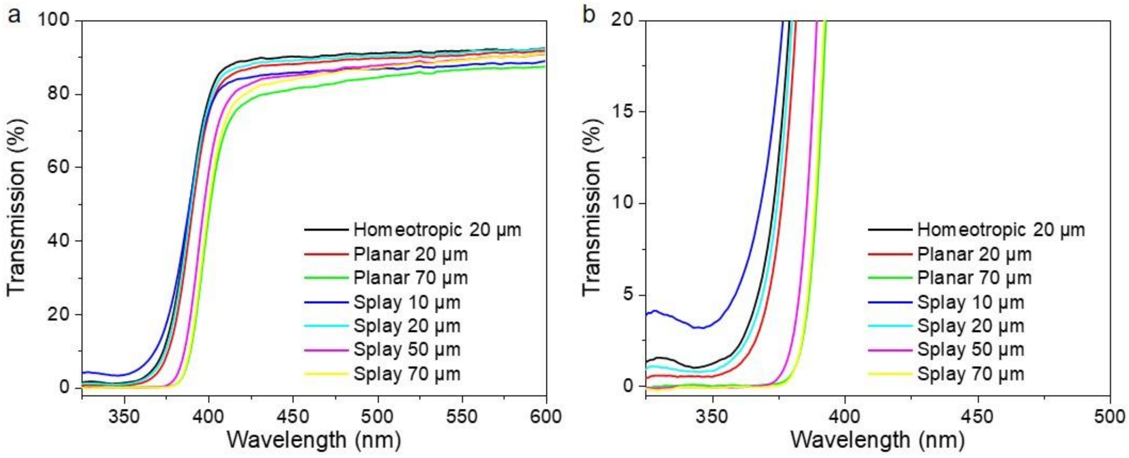

Figure S2. Transmission spectra of the LCN films of various alignments and thicknesses containing 2.5% of Tinuvin **1**.

The heat diffusion equation is given by:

$$\tau = \frac{t^2}{4\alpha} = \frac{\rho t^2 Cp}{4k}$$

Where t is the thickness, α is the thermal diffusivity, k is the thermal conductivity (~ 0.2 $W.m^{-1}.K^{-1}$), ρ is the mass density (1220 $kg.m^{-3}$) and Cp is the specific heat capacity (1050 $J.kg^{-1}.K^{-1}$).
